# Distribution of variations in anatomy of the circle of Willis: results of a cadaveric study of the Malawian population and review of literature

**DOI:** 10.11604/pamj.2021.38.11.27126

**Published:** 2021-01-06

**Authors:** Charles Nyasa, Anthony Mwakikunga, Lackson Tembo, Charles Dzamalala, Amadi Ogonda Ihunwo

**Affiliations:** 1 Biomedical Sciences Department, Anatomy Division, College of Medicine, University of Malawi, Blantyre, Malawi,; 2 Malawi-Liverpool Wellcome Trust Clinical Research Programme, Blantyre, Malawi,; 3 School of Anatomical Sciences, Faculty of Health Sciences, University of the Witwatersrand, Parktown, Johannesburg, South Africa,; 4 Pathology Department, College of Medicine, University of Malawi, Blantyre, Malawi

**Keywords:** Circle of Willis, anatomical variation, neurovascular disease, posterior communicating artery, Osiris

## Abstract

**Introduction:**

the circle of Willis is an anatomical structure of clinical importance particularly in the evaluation of neurovascular diseases. Individuals show considerable variations in the anatomical configuration of the circle of Willis. A cross-sectional study was conducted to determine the distribution of morphological variations of the circle of Willis in Malawians and compare with other ethnic groups.

**Methods:**

brains were collected from twenty-four recently deceased black Malawians during autopsy at Queen Elizabeth Central Hospital, a referral teaching hospital in Blantyre, Malawi and fixed in 10% buffered formalin. Digital images of the interpeduncular region (exposing the circle of Willis) were taken with an 18.4 megapixels camera from the base of the brain. Whole-circle and segmental parameters of the circle of Willis were assessed using the Osiris computer programme and classified based on a 22-type classification scheme.

**Results:**

the following morphological variations were observed: hypoplasia, aplasia, asymmetry and accessory vessels. Typical circle of Willis was seen in 26% of the cases. Only six of the original twenty-two types were observed. Consistent with most previous studies, types 1, 3, 4, 6, 8 and 9 were common while types 10-22 were rare. Three variants not previously described in the original scheme (unilateral PcoA aplasia, AcoA duplication, and PcoA aplasia with contralateral PcoA hypoplasia) were observed in this study.

**Conclusion:**

anatomical variations of the circle of Willis in Malawians seem to be distributed in similar frequencies and patterns as in other more-diverse populations. Circle of Willis variants with potential predilection for atherogenesis and aneurysm formation exist in the Malawian population. These should be considered in clinical practice.

## Introduction

The circle of Willis, also known as the cerebral arterial circle, is a polygonal system of arteries in the interpeduncular cistern at the base of the brain [1]. It is formed by anastomosis of the internal carotid arteries (ICA) and the basilar artery (BA), and serves to allow collateral blood flow and equalization of pressure [2, 3]. The anterior, posterior and middle cerebral arteries; the anterior communicating artery (AcoA) and the posterior communicating artery (PcoA) all contribute to form the circle. Knowledge of the anatomy of the circle of Willis is of great clinical importance [3-5]. Over the years, studies have revealed the highly-variable nature of this structure as observed across sexes, ages and populations [5-8].

The issue of population variations in the anatomy of the circle of Willis is of particular importance as knowledge of their distribution may explain the observed differences in prevalence, progression and pathologic profile of certain neurovascular diseases across geographies [2, 9-13]. Higher frequencies of the variations as well as abnormalities of the circle of Willis are seen to associate with increased risk of cerebrovascular catastrophe [4, 14]. This is no surprise as variations such as aplasia and hypoplasia occurring in certain locations may render the circle ineffective as a collateral channel [15]. For instance, bilateral aplasia of the PcoA may disrupt collateral flow between the anterior and posterior cerebral circulations while aplasia involving both the unilateral PcoA and the AcoA may affect arterial back-up between the right and left ICAs. Using mathematical models, Pascalau *et al*. [3], demonstrated that vessel asymmetry within the circle´s architecture tends to position the flow divider at points of bifurcation unfavourably, leading to turbulence and ignition of endothelial dysfunction. These may play a role in promoting atherogenesis, exacerbating the severity of strokes and favouring formation of aneurysms. Moreover, the risk of intracranial atherosclerosis is reportedly higher among Blacks compared with Caucasians [16], making studies on the anatomy and variations of the cerebral arterial tree among native Africans important in the understanding of neurovascular diseases such as ischaemic stroke.

In 1979, Lazorthes *et al*. described a 22-type scheme for classifying whole-circle anatomic variations of the circle of Willis based on typicality and caliber of component vessels [17]. The scheme has over the years been applied universally to compare the distribution of anatomical variations of the circle of Willis across populations [6, 7, 17]. For instance, El Khamlichi *et al*. applied the scheme in a study of Moroccans [18], De Silva *et al*. of Sri Lankans [19], and Klimek-Piotrowska *et al*. of Poles [7]. To date, it is not clear whether the different varieties of the circle of Willis occur in similar frequencies across racial and ethnic groups as studies have often reported conflicting results [6, 8, 19, 20]. Moreover, the scarcity of whole-circle studies in some populations [20], coupled with differences in protocols and definitions across studies make cross-study comparisons difficult [4, 5, 8]. The present study was conducted to determine the distribution of variations in the anatomy of the circle of Willis among Malawians in comparison with other more-diverse ethnic groups.

## Methods

### Study site and study design

This was a cross-sectional anatomical study conducted in the Anatomy Division, Department of Biomedical Sciences, College of Medicine, University of Malawi. Brains were obtained from a convenient sample of twenty-four recently deceased black Malawians who were candidates for medical-legal autopsies at Queen Elizabeth Central Hospital, Blantyre, Malawi. There were four females and twenty males and their ages ranged from 3 to 65 years. The causes of death for thirteen participants in this study were natural disease processes such as meningitis, pneumonia and hypoglycemia while eleven participants had died of unnatural causes which included poisoning, road traffic accident, electrocution and head injuries related to assault. The study conformed to the World Medical Association Declaration of Helsinki (as revised in Edinburgh 2000) and was conducted in accordance with the Government of Malawi Anatomy Act No. 14 of 1990. Ethical clearance was granted by the University of Malawi´s College of Medicine Research and Ethics Committee (COMREC) with a clearance number P0515/1729. Informed consent was obtained from legally-appropriate guardians or next of kin. The brains were extracted from cranial cavities, washed with isotonic saline to remove blood around the region of interpeduncular fossa, and were fixed in 10% buffered formalin for 48 hours. Cases with remarkable alteration in brain arteries and gross pathological lesions resulting from crush injuries, macroscopically identified tumours or severe haemorrhage were excluded from the study. No participant had a record of any neurologic or neurovascular condition.

### Imaging

Extracted brains were placed on a flat plane having a white background, ventral surface up. Images of the exposed bases were taken with an 18.4 Megapixels camera (Canon EOS 600D, 2011 model, Cannon USA). Before photography, a metric caliper gauge was placed horizontally on the frontal lobes anterior to temporal poles and each image was taken perpendicularly from a 30cm height in order to minimize errors that could have arisen due to variations in angles of view. A similar method was employed by Cilliers *et al*. in a study of South African cadavers [5].

Digital images were initially studied under direct vision to define the whole-circle configuration of the circle of Willis, then processed in the OSIRIS computer programme (University Hospital of Geneva) on a Microsoft Windows 10 computer to measure length and diameter of component vessels. OSIRIS is an open source extensible and portable program for displaying, manipulating and analyzing digital images for research purposes [2]. The use of OSIRIS as a tool for studying the morphometry of cerebral arteries has been described previously [2, 6]. Using the programme, length and diameter values were obtained by measuring pixel intensities along and across (respectively) the length of the vessel followed by scaled conversion of the obtained value to millimeters. Creation of the conversion scale was facilitated by the metric caliper gauge that was included in each image during photography. Images were independently reviewed by three anatomists (CN, AM and LT) who were also part of the study team. Averages of the three independent measurements per vessel segment were calculated for length and diameter, while attributes such as typicality, completeness and presence of accessory vessels were assessed through expert consensus. A mini-analysis of A1 vessel values recorded by the first and second assessors revealed no statistically significant inter-observer variability (paired samples t-test, P = 0.1256 for length and P = 0.9434 for diameter, at a 95% confidence level). In this study, vessels that were targeted included the AcoA, the precommunicating part of ACA (A1), the PcoA and the precommunicating part of PCA (P1). To be consistent with previous studies [2, 7, 19], a cut-off point of 1mm was used to define vessel hypoplasia while symmetry was said to exist when a vessel´s diameter was less than half of its contralateral counterpart.

### Data analysis

Data were processed in Microsoft Excel program and circles of Willis were schematized based on a scheme described by Riggs and Rupp [15]. Descriptive statistics of component vessel properties were run in Stata version 14. The distribution of anatomical variations of the circle of Willis observed in this study was compared with literature from other ethnic groups based on an original 22-type classification system of Lazorthes *et al*. [17]. Comparison of the results is presented only in the form of a table summarizing percentage observations from the literature and the results from this study. Due to the limited nature of the sample size and many zero observations in the present study, the authors could not run robust statistical analysis including hypothesis testing.

## Results

**Figure 1:**
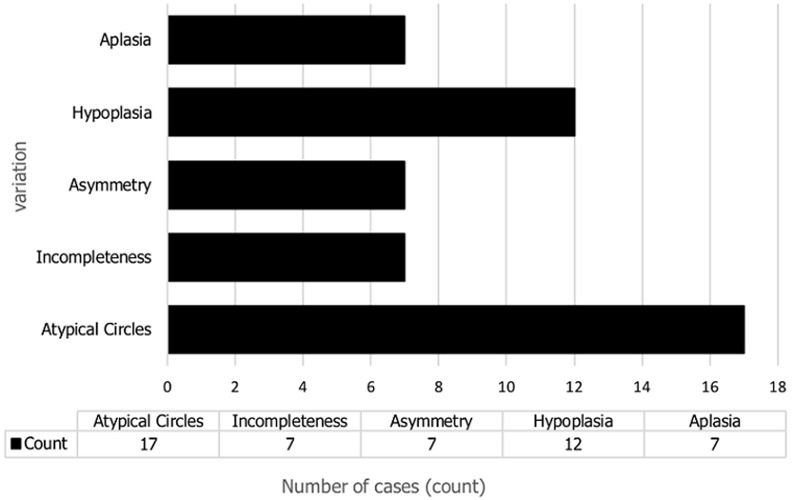
distribution of whole-circle properties and segmental vessel variations observed in this study

In this study, whole circle properties such as typicality, completeness, symmetry of the circle of Willis and vessel parameters such as length and diameter were assessed. One hundred and fifty-eight segmental vessels from the photographed arterial circles were measured and analyzed. Figure 1 outlines a summary of whole-circle properties and segmental vessel variations that were observed in this study.

**Figure 2:**
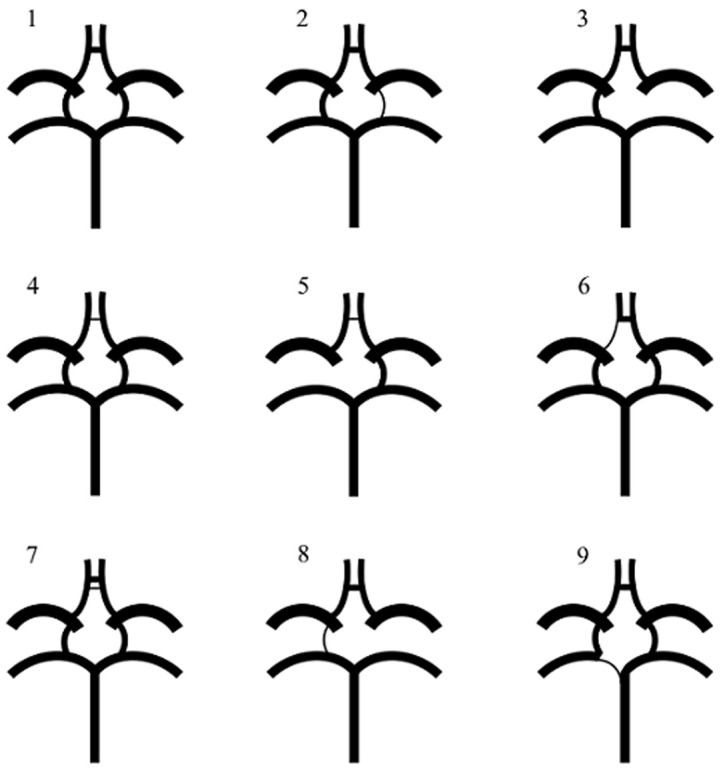
schematic configurations for variants of the circle of Willis observed in this study: (1) all vessels intact (typical variant) n=6; (2) unilateral PcoA hypoplasia n=3; (3) unilateral PcoA aplasia n=6; (4) AcoA hypoplasia n=1; (5) unilateral PcoA aplasia with AcoA hypoplasia n=1; (6) A1 hypoplasia n=1; (7) duplicated AcoA n=1; (8) unilateral PcoA aplasia with contralateral PcoA hypoplasia n=1; (9) P1 hypoplasia n=3 (n = numbers of variants of the circle of Willis)

Eight circle of Willis variants other than the classical text-book (typical) type were observed. Four of these variants involved variations of the PcoA and three involved the AcoA. The A1 and P1 vessels were associated with only one circle of Willis variant each. Of the 24 samples that were studied, a total of 6 circles of Willis exhibited the typical configuration while the rest were atypical. The PcoA was the vessel that was responsible for most of the observed variations, followed by the AcoA and the P 1 (Figure 2).

**Figure 3:**
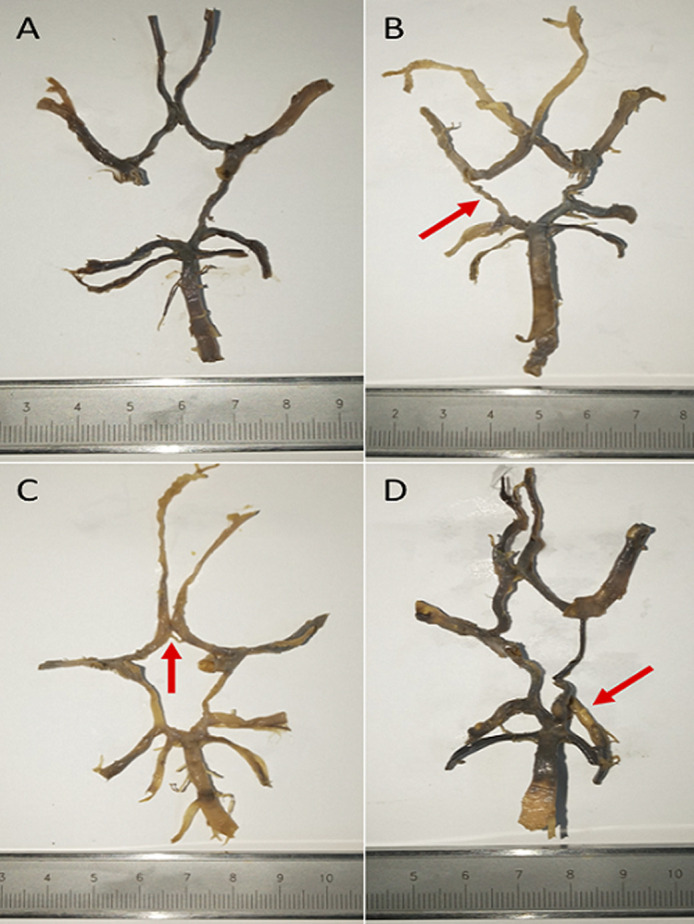
extracted arterial circles showing different variants observed in the present study. (A) unilateral PcoA aplasia; (B) unilateral (right) PcoA hypoplasia (arrow); (C) duplication of AcoA, the proximal element of which was hypoplastic (arrow); (D) typical CoW with calcifications (arrow: glossily visible atherosclerotic plaques

Only 6 of the 22 circle of Willis variants described in the scheme by Lazorthes *et al*. were observed in this study. Type-1 (the typical variant: 26%) was the most common variant, followed by type-4 (Unilateral PcoA hypoplasia: 13%) and type-9 (P1 hypoplasia: 13%) (Table 1). Three variants (unilateral PcoA aplasia: 26.1%, duplicated AcoA: 4.3% and PcoA aplasia with contralateral PcoA hypoplasia: 4.3%) observed in this study had not been previously described in the original classifications. These were classified as ‘other types´ and their configurations were also schematized (Figure 2). Extracted arterial circles exhibiting different variants observed in the present study are also shown in Figure 3.

**Table 1:** summary of comparison of frequencies of the various circle of Willis variants of the present study with other studies

Author and year of study		Present study	Riggs and Rupp (1963)	Fisher (1965)	Lazorthes et al. (1979)	El Khamlichi et al. (1985)	Eftekhar et al. (2006)	De Silva et al. (2011)	Klimek-Piotrowska et al. (2017)	Cilliers et al. (2017)
Country		Malawi	USA	USA	France	Morocco	Iran	Sri Lanka	Poland	South Africa
Total brains (n)		24	994	414	200	100	102	225	100	39
Type	Definition	%	%	%	%	%	%	%	%	%
1	Typical	26.1	19.0	5.0	14.5	18.0	28.0	14.0	27.0	41.0
2	All segmental vessels hypoplastic	0.0	5.0	0.0	5.0	0.0	0.0	0.0	0.0	0.0
3	Hypoplastic AcoA	4.3	9.0	1.0	4.5	11.0	0.0	14.0	2.0	5.1
4	Unilateral hypoplastic PcoA	13.0	9.0	6.0	14.0	14.0	20.0	11.5	11.0	23.1
5	Unilateral hypoplastic PcoA and AcoA	4.3	4.0	3.0	5.0	6.0	4.0	7.0	3.0	5.1
6	Bilateral hypoplastic PcoA	0.0	13.0	32.0	22	24.0	27.0	23.0	16.0	10.3
7	Bilateral hypoplastic PcoAs and AcoA	0.0	7.0	14.0	17	10.0	4.0	16.0	1.0	5.1
8	Hypoplastic A1	4.3	5.0	0.0	1.5	2.0	0.0	3.0	0.0	2.6
9	Unilateral hypoplastic P1	13.0	5.0	0.9	2.5	3.0	0.9	0.8	1.0	0.0
10	Bilateral hypoplastic P1s	0.0	3.0	4.0	3.0	1.0	0.0	0.4	0.0	0.0
11	Hypoplastic P1 and contralateral A1	0.0	0.2	2.0	0.0	0.0	0.0	0.0	0.0	0.0
12	Hypoplastic P1 and ipsilateral A1	0.0	2.0	0.2	1.5	1.0	0.9	2.0	1.0	0.0
13	Bilateral hypoplastic P1s and A1	0.0	0.5	0.7	0.5	0.0	0.0	0.4	0.0	0.0
14	Hypoplastic A1 and contralateral PcoA	0.0	0.7	0.2	0.5	0.0	0.0	0.0	0.0	0.0
15	Hypoplastic AcoA and P1	0.0	3.5	0.0	2.0	4.0	0.9	3.0	0.0	0.0
16	Hypoplastic PcoA, ipsilateral A1 and AcoA	0.0	2.0	0.4	1.0	3.0	0.0	0.0	0.0	2.6
17	Hypoplastic PcoA and contralateral P1	0.0	3.0	11.0	1.5	0.0	2.0	0.4	2.0	0.0
18	Bilateral hypoplastic PcoAs and A1	0.0	6.0	5.0	3.0	0.0	0.0	2.0	0.0	0.0
19	Hypoplastic PcoA, AcoA and contralateral P1	0.0	2.0	7.0	0.5	1.0	0.9	0.0	0.0	0.0
20	Hypoplastic P1, contralateral PcoA and ipsilateral A1	0.0	1.0	1.0	0.0	1.0	0.0	0.0	0.0	2.6
21	Bilateral hypoplastic P1s and AcoA	0.0	1.0	2.0	0.5	0.0	0.0	0.4	0.0	0.0
22	Hypoplastic PcoA, ipsilateral A1 and contralateral P1	0.0	0.3	2.0	0.0	0.0	0.0	0	0.0	0.0
Other	Unclassified variations	35.0	0.5	2.0	0.0	1.0	0.9	2.2	36.0	2.6

## Discussion

### Distribution of variations

All the circles of Willis observed in the present study were schematized and classified based on a 22- type scheme described by Lazorthes *et al*. [17]. The most common variant observed in the present study was type-1 (typical or classical text-book variant of the circle of Willis). This finding supports earlier studies that have reported high prevalence in the typical variant across populations [5, 15-18, 19], where the prevalence higher than 10% was reported. However, this is in variance with the USA study by Fisher that reported a prevalence of 5% [21]. Unlike the rest of the studies in which specimens were collected from representative samples of the general population, Fisher´s study did not select brain samples since it lacked the inclusion and exclusion criteria, which might have included brain tissues with anomalies. This means that generally type-1 variant seems to be the most common anatomical configuration across ethnic groups.

Additionally, the circle of Willis variant exhibiting unilateral hypoplastic P1 (type-9) has been a fairly common finding across populations, although a higher prevalence of 13% was observed in the present study, compared to 5% in USA and Iran, 2.5% in France, 1% in Morocco and Poland, and 0.8% in Sri Lanka [5-7, 15-18, 19] (Table 1). The difference observed between the present study and other studies may be attributed to the small sample size in the present study and the use of proportions as the 13% in this study is representing three brains while the 5% observed in the USA study represents forty-nine brains [15]. Although there was no type-9 case in the South African study [5], other forms of variations involving the same vessel (such as adult, foetal and transitional configuration of the P1-PcoA territory) were observed, thereby reflecting presence of other forms of variations beyond the original Lazorthes classification. It could be speculated that variations of the circle of Willis are distributed similarly across populations. Moreover, types 3 and 8, with a prevalence of 4.3%, were also observed in this study confirming what others have reported [15-18, 19].

Type-6 variant (bilateral hypoplastic PcoAs) also appears to be among the common variants reported in previous studies with the prevalence as high as 27% in Iran, 24% in Morocco, 32% in USA and 10% in South Africa. Although no circle of Willis exhibited this variant in the present study, similar component variations involving the same vessels (the PcoAs), particularly hypoplasia and aplasia (Figure 3), were observed but only unilaterally. Moreover, consistent with all other studies [5-7, 15-1,9], the PcoA was the most variable vessel in the present study. Similarly, type-2 and types 10 to 22 were not observed in the present study reflecting that configurations exhibiting single vessel variations (types 1, 3, 4, 6, 8 and 9) are more common than those with multiple variations (Table 1). In light of the foregoing, it is clear that generally there are similarities in the prevalence and distribution of variations in configuration of the circle of Willis among ethnic groups despite small differences which may be attributed to sample size issues, inconsistent study techniques and embryonic factors during development.

The circle of Willis configurations that were not included in the original classification of Lazorthes *et al*. are not uncommon in populations (Table 1). However, they are commonly reported at low prevalences [7, 15]. Eftekhar *et al*. found one such variant (bilateral hypoplasia of PcoA, unilateral P1 and AcoA) in a study of 102 Iranian cadavers [6]. Similarly, De Silva also reported one such unclassified variant (hypoplastic A1 and contralateral PcoA) among Sri Lankans [19], while Klimek-Piotrowska *et al*. (who unlike most researchers included variations of Superior Cerebellar Arteries (SCA) and terminal portions of the ICA in their classification) found 26 different unclassified variants among Poles [7]. All the three unclassified (or other) variants that were observed in the present study had also been observed in other populations. For instance, variations such as unilateral PcoA aplasia, duplicated AcoA, and PcoA aplasia with contralateral PcoA hypoplasia, bilateral PcoA aplasia and triplicated A1 were observed in Poland [7]. Similar to the present study, cases of duplication of the AcoA were reported in South African population [5]. On overall, these results seem to suggest uniformity in the distribution of circle of Willis variations across populations as all variants (including the unclassified ones) that were seen in the present study were also reported in other diverse populations, although not without some disparities.

### The origin of variations

Regarding the origin of anatomical variations of the circle of Willis, a number of theories have been proposed. Other studies have postulated that variations may result from differences in amplitude of neck movements in later life, haemodynamic factors, postnatal development and genetic factors [6, 22]. Some morphological variations have also been explained on the basis of embryological evolution where cerebral blood vessels are said to develop from primitive plexuses. For instance, during early days of life (between 0.4 mm and 14 mm embryo stages), the ACA, MCA and PCA territories are all supplied by the ICA system [23, 24]. By this stage the PcoAs (which are relatively large is size) branch directly the ICAs through fusion of caudal branches of the ICA with longitudinal neural arteries (the primitive basilar artery) [24-26]. The vertebro-basilar system develops at a later stage to anastomose with the ICA and take over arterial supply of the PCA territory (occipital lobes) from the PcoAs. This anastomosis reduces the ICA field of supply which causes the PcoAs to regress and adopt their adult-form diameter. No wonder, certain forms of variations, such as hypoplastic or aplastic PcoAs which were also observed in the present study have been associated with higher predilection for atherogenesis and aneurysm formation.

The dynamic nature of the evolution of the circle of Willis, anastomosis between the ICA and vertebro-basilar systems in particular, appears to explain the observed higher frequency of variations in the posterior half of the circle in comparison with the anterior half [2, 11, 22]. Excessive regression of the PcoA vessels at this stage has been associated with hypoplasia in later life while persistence has been associated with occurrence of the fetal circle of Willis variant in which case, PcoAs tend to be wider than the precommunicating part of the PCA.

Similar to Eftekhar *et al*. [6], the results of this study do not seem to suggest that factors responsible for variations of the circle of Willis are different in different populations or act differently. The uniformity in the distribution of variations that has been observed in this and other studies rather appears to support the role of embryological evolution where variations ensue in response to differences in physiological demands of various parts of the brain as dictated by patterns of fetal development which are fairly uniform among all humans. Saikia *et al*. suggested that the variation of circle of Willis may be attributed to triggers at various stages of embryonic development [26]. According to these researchers, triggers are a phenomenon that may alter vascular construction programme either in a transient or in a permanent way [26]. Similarly, Lazorthes *et al*. thought the variations are due to progressive modelling in response to differences in demands for blood supply during prenatal life [5, 17]. Other factors (genetic and environmental) may also play a role in giving rise to or modifying these variations, and this is an area of on-going research.

### Limitations of the study

The operator-dependent nature of the computer-assisted measurement method employed in this study (as is common across all morphometric cadaveric studies) may limit the exactness of segmental values that were used to define vessel asymmetry, hypoplasia and typicality. However, a mini-study conducted in the A1 vessel revealed no statistically significant differences in length and diameter between first and second measurements in this study. Some of the vessels in this study appeared to be under traction and attempts were not made to decompress these collapsed vessels. Additionally, vessel diameter was measured at the seemingly widest spot. In this case, it was assumed that diameter along a length of a vessel was uniform. It cannot be commented on whether this spot represented the true diameter of the whole vessel. Ansari *et al*. used a similar system [2], while Hoksbergen *et al*. [14], used the narrowest part of the artery for analysis as they believed that this part determined collateral ability of the entire vessel.

## Conclusion

This study has described the circle of Willis variants that exist in the Malawian population for the first time. The pattern and frequency of variations observed in this study appear to be similar to those reported in other diverse populations. Therefore, these results seem to suggest that anatomical variations of the circle of Willis are distributed similarly across populations. Circle of Willis variants with potential predilection for atherogenesis and aneurysm formation appear to exist in the Malawian population. This knowledge will help clinicians and researchers understand anatomical bases of certain neurovascular diseases.

### What is known about this topic

- Variations in morphology of the circle of Willis exist in the world´s population;
- Variations of the circle of Willis have been linked to incidence and progression of neurovascular diseases including stroke and aneurysms;
- Studies on the anatomy of the cerebral circulation and its variations in the sub-Saharan African population, including Malawi, are very rare.

### What this study adds

- Variations in morphology of the circle of Willis exist in the world´s population;
- Variations of the circle of Willis have been linked to incidence and progression of neurovascular diseases including stroke and aneurysms;
- Studies on the anatomy of the cerebral circulation and its variations in the sub-Saharan African population, including Malawi, are very rare.
